# The impact of COVID-19 vaccination on California’s return to *normalcy*

**DOI:** 10.1371/journal.pone.0264195

**Published:** 2022-05-19

**Authors:** Maria L. Daza–Torres, Yury E. García, Alec J. Schmidt, Brad H. Pollock, James Sharpnack, Miriam Nuño

**Affiliations:** 1 Department of Public Health Sciences, University of California Davis, Davis, CA, United Sates of America; 2 Department of Statistics, University of California Davis, Davis, CA, United Sates of America; South African Medical Research Council, SOUTH AFRICA

## Abstract

SARS-CoV-2 has infected nearly 3.7 million and killed 61,722 Californians, as of May 22, 2021. Non-pharmaceutical interventions have been instrumental in mitigating the spread of the coronavirus. However, as we ease restrictions, widespread implementation of COVID-19 vaccines is essential to prevent its resurgence. In this work, we addressed the adequacy and deficiency of vaccine uptake within California and the possibility and severity of resurgence of COVID-19 as restrictions are lifted given the current vaccination rates. We implemented a real-time Bayesian data assimilation approach to provide projections of incident cases and deaths in California following the reopening of its economy on June 15, 2021. We implemented scenarios that vary vaccine uptake prior to reopening, and transmission rates and effective population sizes following the reopening. For comparison purposes, we adopted a baseline scenario using the current vaccination rates, which projects a total 11,429 cases and 429 deaths in a 15-day period after reopening. We used posterior estimates based on CA historical data to provide realistic model parameters after reopening. When the transmission rate is increased after reopening, we projected an increase in cases by 21.8% and deaths by 4.4% above the baseline after reopening. When the effective population is increased after reopening, we observed an increase in cases by 51.8% and deaths by 12.3% above baseline. A 30% reduction in vaccine uptake alone has the potential to increase cases and deaths by 35% and 21.6%, respectively. Conversely, increasing vaccine uptake by 30% could decrease cases and deaths by 26.1% and 17.9%, respectively. As California unfolds its plan to reopen its economy on June 15, 2021, it is critical that social distancing and public behavior changes continue to be promoted, particularly in communities with low vaccine uptake. The Centers for Disease Control and Prevention (CDC) recommendation to ease mask-wearing for fully vaccinated individuals despite major inequities in vaccine uptake in counties across the state highlights some of the logistical challenges that society faces as we enthusiastically phase out of this pandemic.

## Introduction

The coronavirus pandemic has highlighted how inadequate and unprepared the public health and healthcare system was for such an event, with disproportionate consequences for traditionally underserved populations. As of May 19, 2021, the US had administered at least one dose of available vaccine to nearly 48% of the adult population, with 37.8% fully vaccinated. California has used 79% of its vaccine supplies to fully vaccinate 47.8% of the adult population, ranking 27th, proportionally, out of all states.

Management of the pandemic at the county level has thus far been lead by California’s *Blueprint for a Safer Economy* [1], which uses local case and test positivity rates, adjusted for equity measures in the most vulnerable census tracts, to determine a color-based qualitative threat level for the entire county. Business operation, non-pharmaceutical intervention (NPI) mandates, and general advice on safety and maximum gathering sizes are tied to these threat levels. In the recent *Beyond the Blueprint for a Safer Economy*, The California Department of Public Health (CDPH) issued plans and guidance for a full easing of all business restrictions by June 15th, 2021, contingent on case, vaccination, and equity measurements staying on track. CDPH stressed the importance of maintaining infrastructure and resources for continuing vaccination programs; monitoring for new cases and strains with active testing, especially in the most vulnerable areas of the state; robust contact tracing and outbreak investigations; statewide plans to scale up resources in the case of another large outbreak; and monitoring hospital usage, appropriate availability of personal protective equipment, and surge capacity [2].

It is unlikely that any given state in the US will be able to eliminate SARS-CoV-2 completely. Short of elimination, the next best hope is that forcing the virus into endemicity will result in a much less severe disease in years to come [3], which is the foundation for California’s current approach. For infectious diseases like COVID-19, the risk and size of an outbreak—and the threshold for forced endemic transmission—is determined by the transmissibility of the virus and the effective size of the population who can acquire it. Transmissibility is a function of biochemical, biological, and social factors and can thus change over time in unpredictable ways. Multiple new strains of the SARS-CoV-2 virus have already appeared over the course of this pandemic, the interactions of which with natural and pharmaceutical immunity is only partially explored [4]. Though acquiring an infection after a complete vaccination regimen is rare [5, 6], the continued appearance of new strains, the risk of which increases with circulation around the world, could reduce vaccine effectiveness [7, 8]. Additionally, messaging around the lifted restrictions and pandemic fatigue may lead people to change their behaviors in ways that could change transmission rates, such as ignoring mask use, social distancing, and limitations on indoor gatherings of unvaccinated individuals [9, 10].

Traditional Susceptible-Exposed-Infectious-Removed (SEIR) models with fixed model parameters tend to be too simplistic or divergent from real-world data to act as good assessments of future trends to form the basis of an exit strategy. These models assume homogeneous mixing in a population, that is, all individuals are equally likely to encounter an infected individual and that their behavior remains constant throughout the period of study. This assumption is sensitive to people’s contact patterns and behavioral changes that impact infection risk. We address this limitation by implementing a modeling framework that allows for the estimation of critical parameters, and the updating of these parameters (e.g. the effective population proportion that is the proportion of people with a high probability of having contact with an infected person due to their behavior.) to improve our understanding of disease spread. This approach involves a Bayesian sequential data assimilation model that allows for non-stationarity by sequentially updating model parameters, such as transmission rates and the effective population. In this way, our model can implicitly account for the unobserved changes in social distancing behavior, mobility, masking behavior, etc. We used posterior coverage intervals to investigate the uncertainty of and establish reasonable ranges for these model parameters to create realistic scenarios for widescale re-opening and the lifting of restrictions. We summarize the potential impact of changes in transmissibility, effective population, and vaccination rates by observing how they can affect incident cases and deaths in the weeks after the easing of restrictions in California.

## Materials and methods

### Mathematical model

**Fig 1:**
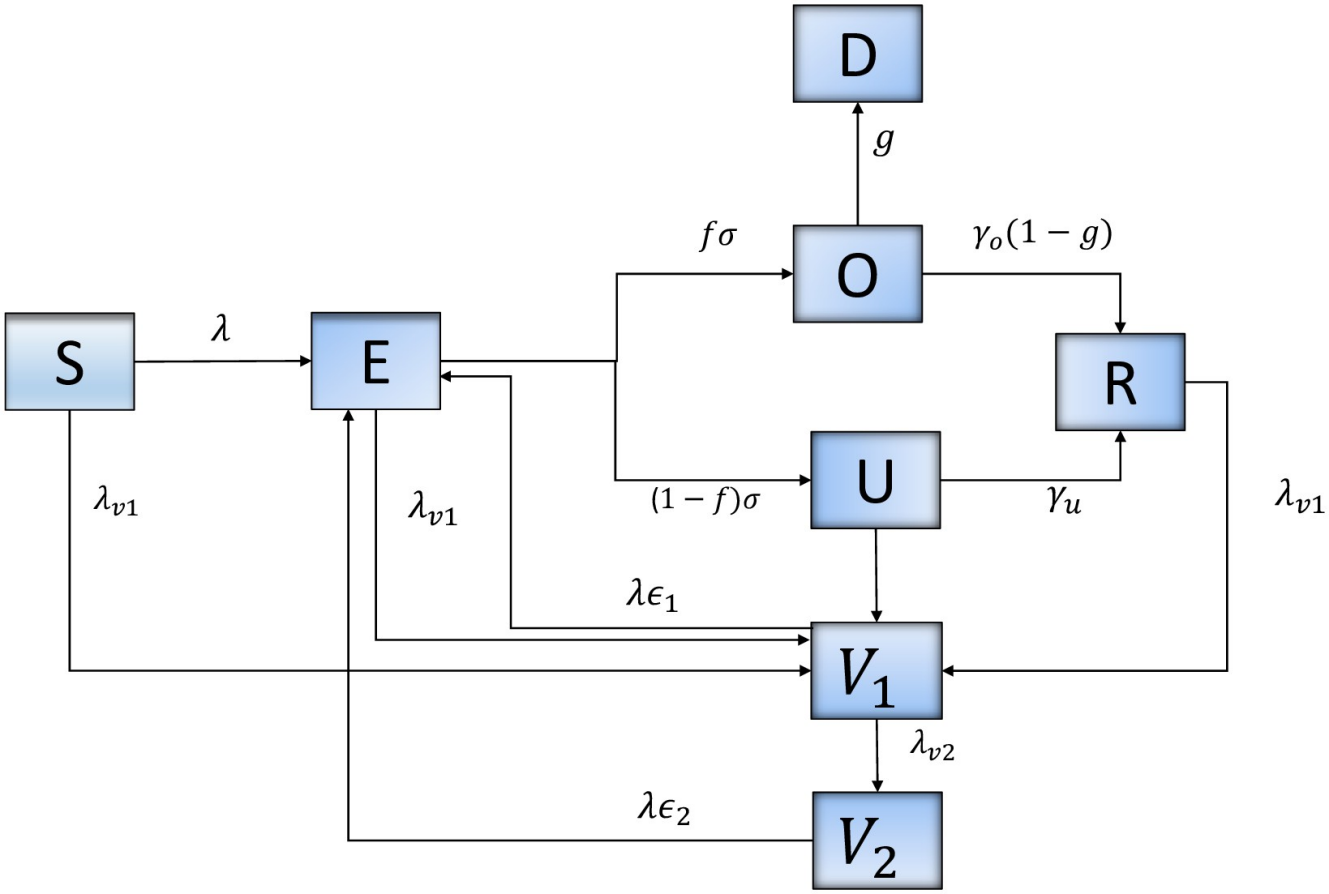
Flow diagram of SARS-CoV-2 transmission dynamics with vaccination.

A dynamic SARS-CoV-2 transmission model with vaccination was implemented (Fig 1). The total population was divided into eight classes: susceptible, exposed, reported infectious, unreported contagious, vaccinated, recovered, and deceased. Individuals in the susceptible (S) group become infected and move to the exposed group with the incubation of the virus. Exposed (E) individuals subsequently move to the reported infectious group (O) or the group of unreported contagious (U). Individuals move to compartment *V*_1_ after receiving the first vaccine dose and move from *V*_1_ to *V*_2_ when fully vaccinated. Lastly, individuals are removed through recovery (R) or COVID-19 induced death (D). We assume that recovered individuals acquired immunity for a minimum of two months, in this study this corresponds to the period between May 19 to July 15, 2021. Given limited evidence about the risk of reinfection within a short period of time from infection, our model did not considered this possibility. Recent studies suggest that immunity against reinfection is expected to last 3 to 61 months after developing COVID-19 [11–13].

### Model structure

A dynamic model of SARS-CoV-2 transmission was developed based on eight epidemiological classes as illustrated in Fig 1. We model the process of vaccination by considering that: (1) individuals in all epidemiological classes, with the exception of those diagnosed as positive are vaccinated, (2) vaccine is partially effective, suggesting that some individuals vaccinated can be infected, (3) vaccine efficacy is different after first and second dose administration, and (4) vaccination rate is estimated using daily data of first and second doses administered in California.

$$\begin{array}{c} \begin{array}{cc} \dot{S} & =-\lambda S-\lambda_{v_{1}}S\\ \;\dot{V_{1}} & =\lambda_{v_{1}}\left(S+E+U+R\right)-\lambda_{v_{2}}V_{1}-\lambda \epsilon_{1}V_{1}\\ \dot{V_{2}} & =\lambda_{v_{2}}V_{1}-\lambda \epsilon_{2}V_{2}\\ \dot{E} & =\lambda S-f\sigma E-\left(1-f\right)\sigma E-\lambda_{v_{1}}E+\lambda \epsilon_{1}V_{1}+\lambda \epsilon_{2}V_{2}\\ \dot{O} & =f\sigma E-\left(1-g\right)\gamma_{o}O-gO\\ \dot{U} & =\left(1-f\right)\sigma E-\gamma_{u}U-\lambda_{v_{1}}U\\ \dot{R} & =\left(1-g\right)\gamma_{o}O+\gamma_{u}U-\lambda_{v_{1}}R\\ \dot{D} & =gO \end{array} \end{array}$$ (1)

We assume that only a small proportion of individuals diagnosed as positive contribute to new infections because they do not follow recommendations to isolate. We assume that asymptomatic individuals can also be infectious, so $\lambda =\beta \frac{\left(U+kO\right)}{\omega N}$. *N* = *S*(*t*) + *V*_1_(*t*) + *V*_2_(*t*) + *E*(*t*) + *O*(*t*) + *U*(*t*) + *R*(*t*) + *D*(*t*) corresponds to the total population of California. Parameter description and values are summarized in Table 1.

**Table 1:** Parameter definition and estimates.

**Parameter**	**Description**		
*β*	Transmission rate	Estimated	
*ω*	The effective population proportion	Estimated	
λ_*v*1_	Vaccination rate, first dose	Estimated	
λ_*v*2_	Vaccination rate, second dose	Estimated	
*g*	Mortality rate	Estimated	
**Parameter**	**Description**	**Value**	**Ref**.
*κ*	Proportion of observed people that contribute to new infections	0.2	
*f*	Proportion of observed individuals	0.6	
*N*	California population size	39512223	[15]
1/*γ*_*o*_	Average time to recovery, diagnosed	1/14	[16, 17]
1/*γ*_*u*_	Average time to recovery, undiagnosed	1/7	[18]
*ϵ*_1_	(1 − *ϵ*_1_) Vaccine efficacy after first dose	0.40	[19]
*ϵ*_2_	(1 − *ϵ*_2_) Vaccine efficacy after second dose	0.05	[19]
*σ*	Median number of days from symptom onset	1/5	[20]

### Parameter estimation

The Bayesian sequential forecasting method in Daza-Torres, et al. [14] is used to conduct parameter inference. The estimation is implemented by decoupling the model into two parts. First, we consider the transitions for vaccination [Eq 3] and second, the remaining dynamics [Eq 2].

$$\begin{array}{c} \begin{array}{cc} \dot{S} & =-\lambda S\\ \dot{E} & =\lambda S-f\sigma E-(1-f)\sigma E\\ \dot{O} & =f\sigma E-\gamma_{o}O-\left(1-g\right)\gamma_{o}O\\ \dot{U} & =\left(1-f\right)\sigma E-\gamma_{u}U\\ \dot{R} & =\left(1-g\right)\gamma_{o}O+\gamma_{u}U\\ \dot{D} & =\gamma_{o}gO \end{array} \end{array}$$ (2)

Data on weekly moving average of confirmed cases and deaths are used to estimate the contact rate (*β*), the proportion of the effective population (*ω*), the fraction of individuals infected that are deceased (*g*), and the initial conditions for all compartments, except for the susceptible ones which is set as *S*(*t*_0_) = *ω* ⋅ *N* − (*E*(*t*_0_) + *O*(*t*_0_) + *U*(*t*_0_) + *R*(*t*_0_)) + *V*_1_(*t*_0_) + *V*_2_(*t*_0_).

Let *W* be the population of non-vaccinated individuals at time *t*. For *t*_0_ = 0, *W*(*t*_0_) = *N*. We assume a vaccination rate, for first and second doses to be constant and proportional to the actual population. Therefore,

$$\begin{array}{c} \begin{array}{cc} \dot{W} & =-\lambda_{v_{1}}W\\ \dot{V_{1}} & =\lambda_{v_{1}}W-\lambda_{v_{2}}V_{1}\\ \dot{V_{2}} & =\lambda_{v_{2}}V_{1} \end{array} \end{array}$$ (3)

Data on individuals with at least one dose and fully vaccinated is required to find the value of $\lambda_{v_{1}}$ and $\lambda_{v_{2}}$.

### Scenarios

We propose a set of scenarios to analyze the impact of reopening on June 15th, considering changes in vaccination rate, virus transmission rate, and the effective population proportion. Variability in the rate of infection can inherently reflect the use of masks and new strains of the circulating virus. Changes in the effective population proportion implicitly capture the number of people who are available to be infected due to restrictions or openings.

For the baseline scenario, we fitted the model to updated data until May 18, 2021, and we projected total positive cases and deaths from May 19 through July 15, 2021. Starting on June 15th (reopening day), simulations were evaluated with two different values for both transmission rate (*β*) and the effective population proportion (*ω*). Other parameters were the same as those estimated in the baseline scenario. Replications were evaluated for situations where the vaccination rate was reduced or increased by 30%. Table 2 summarizes the scenario assumptions.

**Table 2:** Summary of scenarios.

	Vaccination rate assumptions		
Description	Current rate maintained	Current rate reduced by 30%	Current rate increased by 30%
Assumption: the viral transmission rate changes after June 15th.	*β*_1_ = 0.4	*β*_1_ = 0.4	*β*_1_ = 0.4
	*β*_2_ = 0.5	*β*_2_ = 0.5	*β*_2_ = 0.5
Assumption: the effective population proportion changes after June 15th.	*ω*_1_ = 0.3	*ω*_1_ = 0.3	*ω*_1_ = 0.3
	*ω*_2_ = 0.5	*ω*_1_ = 0.5	*ω*_1_ = 0.5

Changes in vaccination rates are paired with changes in the effective population proportion (*ω*) or the transmission rate (*β*). Model for the baseline scenario is fitted using updated data through May 18, 2021, generating parameters as in Table 1.

## Results

**Fig 2:**
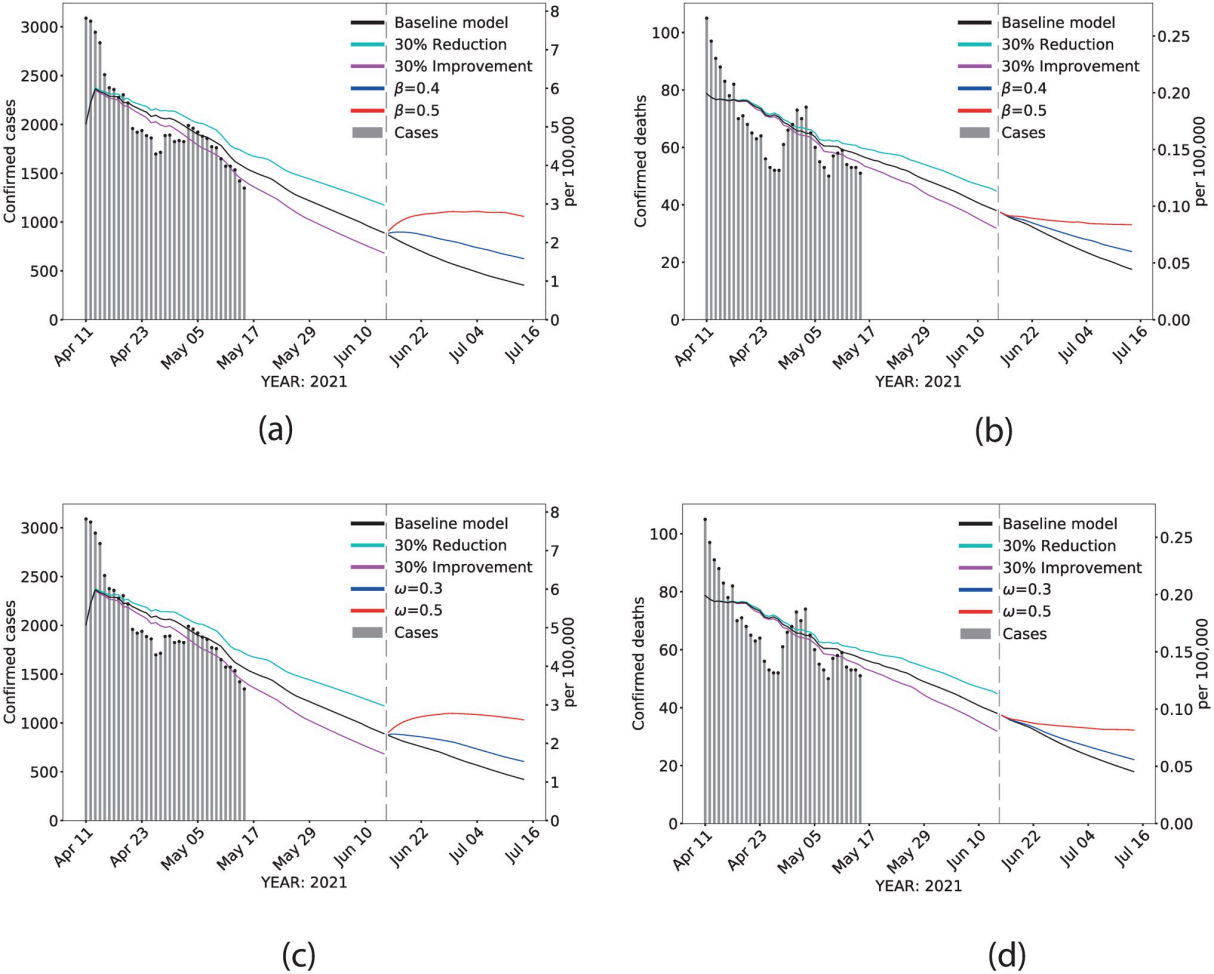
Scenarios. Estimated statewide confirmed cases and confirmed deaths during live data collection, extrapolated between May 18th and June 15th for different vaccination rates, and predicted beyond June 15th with different effective population proportions and transmission rates. (A) and (B): effects of varying transmission rate on confirmed cases and confirmed deaths, respectively. (C) and (D): effects of varying the effective population proportion on confirmed cases and confirmed deaths, respectively. Dashed vertical line: June 15th. Gray vertical bars: daily reported data. Black line: baseline scenario, before and after opening. Cyan line: projection from May 18th assuming a 30% reduction in the current vaccination rate. Magenta line: projection from May 18th assuming a 30% increase in the current vaccination rate. The values used for *β* = 0.4, 0.5, and *ω* = 0.3, 0.5 were selected according to historical data in CA (see S6 Fig in S1 File).

Daily confirmed infections and deaths were projected through July 15, 2021. Fig 2 depicts the predicted daily cases and deaths for California after reopening day. The black line corresponds to the baseline scenario, the cyan line corresponds to the projection assuming a 30% reduction in the current vaccination rate, and the magenta line corresponds to the projection assuming a 30% increment in the current vaccination rate. After June 15, the red, blue, and black lines correspond to the projection with the different values of *β* (Fig 2A and 2B) and *ω* (Fig 2C and 2D). Assessing the impact of different rates of transmission (*β*) allows for flexibility in changes in transmission resulting from reduction in social distancing measures, mask-wearing, and uncertain circulation of new strains after reopening. We further considered scenarios that allow for variability in the effective population proportion (*ω*), as it is likely that reopening will result in increased social interactions with unvaccinated, susceptible individuals. Parameter assumptions for the rate of transmission and the effective population proportion were estimated from California’s own pandemic trajectory (see S6 Fig in S1 File).

### Vaccine and transmission rate effects on projections

After June 15 and assuming current vaccine uptake (*Baseline model*), confirmed cases in California approached 1 case per 100,000 population and 0.03 deaths per 100,000 population (Fig 2A and 2B). Increasing transmission rate to 0.5, a value that reflects the highest peak experienced in California, has the potential to double cases and deaths from baseline estimates. Table 3 provides the estimates of confirmed cases and deaths assuming changes in vaccination rates, transmission rates, and the effective population proportion. Assuming that the current vaccine uptake remains consistent with the current trend but we assume a transmission rate similar to the one observed during California’s highest peak, we project a 48.5% and 9.6% increase from baseline in cases and deaths, respectively, 15 days after reopening. Simultaneously increasing the rate of transmission to 0.4 and reducing vaccine rate by 30% increases the percentage change in cases by 65.7% compared to baseline. Increasing vaccination rate by 30% from the current trajectory has the potential to reduce cases (-10.1%) and deaths (-14.6%) even under a rate of transmission that is higher than baseline (*β* = 0.31).

**Table 3:** Parameters values for the baseline scenario correspond to the posterior median value *β*_*base*_ = 0.31, *ω*_*base*_ = 0.12, λ_*v*1_ = 0.00598, λ_*v*2_ = 0.032; *β* = 0.4, 0.5, and *ω* = 0.3, 0.5 were selected according to historical data in CA (see S6 Fig in S1 File).

	Vaccination Rate Assumptions					
	Current rate maintained	Current rate reduced by 30%	Current rate increased by 30%	Current rate maintained	Current rate reduced by 30%	Current rate increased by 30%
Parameters	Increase or prevention percentage in cases 15 days after opening			Increase or prevention percentage in deaths 15 days after opening		
*β*_*base*_ = 0.31	11429*	35.0	-26.1	429*	21.6	-17.9
*β*_1_ = 0.4	21.8	65.7	-10.1	4.4	27.8	-14.6
*β*_2_ = 0.5	48.5	103.4	8.4	9.6	36.8	-10.8
*ω*_*base*_ = 0.12	9829*	34.0	-25.0	402*	23.5	-16.4
*ω*_1_ = 0.3	51.5	90.5	22.7	12.3	35.8	-5.1
*ω*_2_ = 0.5	68.9	108.2	37.6	15.6	38.8	-2.7

*Base scenario values (total cases and deaths between June 15 and June 30, 2021). All percentages are calculated based on these values.

### Vaccine and effective population effects on projections

Projections of confirmed cases and deaths post June 15 that consider scenarios in which the effective population proportion could increase due to removal of restrictions, resulting in gatherings of susceptible unvaccinated individuals exhibits higher cases and deaths, and much slower downward trends over the period of projection (Fig 2C and 2D). Increasing the effective population proportion from the baseline of 0.12 to 0.3 resulted in a 51.5% and 12.3% increase in cases and deaths, respectively; these estimates were further increased to 90.5% for cases and 35.8% for deaths with a 30% reduction in the current vaccine uptake.

## Discussion

Our data-driven models produced projections of COVID-19 cases and deaths following the reopening of California’s economy. The real-time Bayesian data assimilation modeling approach provides a more realistic method that leverages historical data. This model takes into account time dependence of contact rates, the effective population proportion, and vaccination rates resulting from the implementation of containment strategies and other factors that have fluctuated throughout this pandemic. We investigated several scenarios to provide insight into the effects of the potential consequences of easing mask-wearing restrictions, decreased social distancing, and the growing threat of coronavirus variants in the changing landscape of vaccine uptake. The baseline scenario in which vaccination rate, transmission rate, and the effective population proportion stays roughly constant through July, and we found that state-aggregate case and death rates will steadily decrease. However, if removal of restrictions leads to increases in transmission rate or increases in the effective population proportion, then the current decreasing trend of case rates and death rates is liable to reverse itself, and these rates will persist at a low level. This is especially true if vaccination rates continue to slow from estimates in May. A combination of increase in transmission rates (or the effective population proportion) due to relaxing mitigation measures and a decrease in vaccination rates will likely lead to another surge of cases.

The proposed model assumes that the effects of interventions on infection transmission benefit the population overall. However, COVID-19 has disproportionately impacted vulnerable populations, and efforts to reduce the burden among these groups should be magnified. State-level plans to return to normalcy should involve comprehensive levels of vaccine uptake and transmission reduction measures that consistently and equitably meet the needs of all Californians, with targeted efforts that will improve health and reduce the risk of those at greater risk of severe COVID-19.

Based on data from the current pandemic and information on other related betacoronaviruses, the current dominant strains of SARS-CoV-2 are on track to ultimately become a mild, endemic disease [3]. Unfortunately, that process could take years. A steady but low rate may not initially draw much concern, but the aggregate nature of such data (and derived predictions) can hide some important dynamics. Vaccines have not been equitably dispensed across the state [21], and some counties have consistently had higher case and death rates than others. A stable observed aggregate could very well hide a local outbreak, especially in chronically underserved areas with less robust disease surveillance capabilities. The greater the number of outbreaks and the greater their severity, the more likely a new strain is to emerge.

We assumed that neither natural immunity nor pharmaceutical immunity waned over the analysis window, meaning someone who recovered in April will not have lost any protection by the middle of July. While this fits observations that reinfection is rare in the months after recovery [22], longer-term natural immunity is still unknown. While antibodies for similar betacoronaviruses, such as SARS-CoV-1, become undetectable within a matter of months, T cells remain detectable for a decade or more [23]. There may also be significant cross reactivity with other betacoronaviruses, including the four human coronaviruses that cause the common cold [23, 24], though the SARS viruses seem to create a more specific secondary immune response than the other betacoronaviruses tested [23]. Developing this long-lasting immune response from exposure to a mild disease in childhood can lead to low-impact infections in old age, even if the disease continues to spread [3].

Any exit strategy that aims to push an active infection into an endemic state must account for the possibilities discussed here. There are still concern and speculation around the world as to why the transmission rate remains high despite successful vaccine rollouts in places like Chile [25], the rising cases among young children [26], and the severity increase in young people [27].

Active monitoring of the situation will be required beyond the date of opening, possibly for years to come.

This work represents the forecast of a dynamic model with eight epidemiological classes and vaccination that aims to simulate the disease transmission process to provide projections of cases and deaths in California after the reopening of its economy. We have demonstrated the potential of a Bayesian data assimilation method to capture the temporal evolution of the parameters involved in the transmission model through a trade-off between the complete history of the outbreak and its latest behavior. This model allows us to capture changes in human behavior, virus dynamics, and restriction measures through the time dependence introduced into the parameters involved in the model each time the prediction is updated. This method uses a data window that moves each time new data is updated using the same number of data for each forecast as well as the number of parameters to estimate. We found that the contact parameters and the effective population proportion are the most critical parameters that influence the projections of cases and deaths, post reopening.

### Limitations

There are several limitations within our modeling framework that are important to address. Our models do not explicitly capture forms of social influence and individual level behavior which may influence virus spread. Our study provides global estimates for cases and deaths for California overall and is limited to projections at the county level. Future studies are needed to understand county by county variability in vaccine uptake rates and how these impact incident cases and deaths associated with COVID-19. Our model assumed homogeneous host mixing which assumes that all participants have identical rates of contacts leading to disease transmission. To mitigate the restrictiveness of this assumption, we included the effective population proportion parameter to our model, which allows for added flexibility in our assumed proportion of individuals susceptible to being infected due to different restrictions, openings, and social behavior. In addition, our model is time dependent allowing the estimated model parameters to vary according to historical data. However, our current model is unable to fully capture the dynamics with specific or localized restrictive measures, or super-spreader events. Another limitation is the absence of age and other risk factors, such as comorbidities, that may impact both infections and hospitalizations. Some of these aspects can be included, as well as more detailed transitions of the dynamics of the virus. However, for practical purposes, our transmission model has made a large number of simplifying assumptions mostly driven by the inability to access data with the appropriate spatio-temporal resolution and coverage. Another limitation in our model is our assumption of the infectivity rate, although currently based on historical data for California, it is likely that it is dynamically changing over time as new variants arise. Another important limitation in this study pertains to the quality and availability of the data used. The reliability of the projections made by our model depends on the quality of the input data. Our model uses both records of confirmed cases and deaths of COVID-19 due to a belief that the records of deaths are less subject to sampling bias. The case count data depends on the test protocol used in each locality. In most cases, the tests are performed when symptoms appear, introducing a bias due to the growing evidence that asymptomatic individuals are infectious and individuals who eventually become symptomatic and infectious before the onset of any symptoms. In our model, we postulate a value for the proportion of observed and unobserved infectious that depends on the local practices of applying tests and data reporting.

## Supporting information

S1 File (PDF)
