# Supplementary material for: The impact of COVID-19 vaccination on California’s return to normalcy
Source: PLoS One. 2022 May 19;17(5):e0264195. doi: 10.1371/journal.pone.0264195 (PMC9119543; doi:10.1371/journal.pone.0264195)
Supplement: S1 File — (PDF) [file pone.0264195.s001.pdf]

The impact of COVID-19 vaccination on California's return to *normalcy*

Maria L. Daza–Torres[1‡], Yury E. García [1‡], Alec J. Schmidt [1‡], Brad H Pollock[1‡], James Sharpnack [2‡], Miriam Nuño [1‡],

1 Department of Public Health Sciences, University of California Davis, CA, USA
2 Department of Statistics, University of California Davis, CA, USA

‡These authors also contributed equally to this work. * mdazatorres@ucdavis.edu; mnuno@ucdavis.edu.

List of Supplementary Materials

S1 Daily average vaccination rates in California
Figures S1-S6
Tables S1-S2
S2 Bayesian analysis
S3 Other results

S1 Daily average vaccination rates in California

The average weekly doses of COVID-19 vaccines administered in California have been decreasing since April 11, 2021, coincidentally the week with the highest number of doses administered (Fig S1). In April 11, 2021, the average daily total doses of vaccine administrated were 400,358. The daily average in May 18, 2021, was 222,218. This represents a 44.5% reduction in vaccine uptake compared with the highest rate documented. In the week of April 11, a daily average of 253,785 new doses and 211,898-second doses were administered. In May 18, 2021, the average of first doses administrated decreased by 64% and second doses decreased by 34%, compared to the vaccine rates on April 11. Based on this vaccination trajectory for California, we proposed scenarios on the reduction and uptake of vaccination rates of 30%.

S2 Bayesian analysis

To conduct parameter estimation, we work with a decoupled model, explained below. Once the parameters are estimated, the model described in the main text (Eq 1) is used to simulate the different scenarios.

S2.1 Vaccine coverage model

To model future vaccination coverage, we proposed a compartmental model that includes the dynamics between the not vaccinated population, those who got the first vaccine dose and those who are fully vaccinated. Let W be the not vaccinated people at time t. We assume that no vaccines have been administered at $t = 0$, which implies $W(0) = N$. Then, by assuming that we vaccinate individuals at a constant rate for both

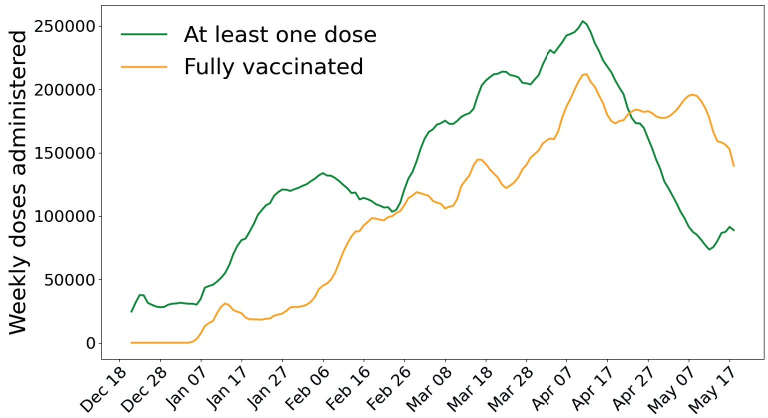

Fig S1. Moving average of administrated doses in California. We use a 7-day moving average to visualize the number of administrated doses. This average is calculated for each day by averaging the values of that day and the six days before. This approach helps prevent significant events (such as changing reporting methods) from skewing the data.

first dose and second dose proportional to the current population, we have $W(t)$, $V_1(t)$ and $V_2(t)$ that satisfy,

$$\begin{aligned} \dot{W} &= -\lambda_{v_1} W \\ \dot{V_1} &= \lambda_{v_1} W - \lambda_{v_2} V_1 \\ \dot{V_2} &= \lambda_{v_2} V_1 \end{aligned} \tag{1}$$

where $W(0) = N$, $V_1(0) = 0$, and $V_2(0) = 0$. We stored the cumulative vaccinated population with at least one dose as:

$$\dot{A} = \lambda_1 W.$$

Since real-world vaccination rates (λ_{v_1}, λ_{v_1}) have changed since the start of the vaccination, we used the prior 30 days of real-world vaccination data to adjust our model rates. Information on fully vaccinated people and people with at least one dose is required to find the value of λ_{v_1} and λ_{v_2}.

S2.1.1 Observational model and data

The observed data used to fit the model (1) are based on the records of people with at least one dose and people fully vaccinated. We consider daily data from the first dose of vaccines administered a_i and its theoretical expectation that is estimated in terms of the dynamical model as

$$\mu_A = A(t_i) - A(t_{i-1}).$$

Analogously, we consider daily second doses administered u_i, and its theoretical expectation given by

$$\mu_{V_2} = V_2(t_i) - V_2(t_{i-1}).$$

S2.1.2 Estimation of model parameters with MCMC

To carry out a likelihood-based analysis, we postulate that the number of both at least one dose and fully vaccinated doses administered follows a Poisson distribution, *Pois*. For data, y_i, we let

$$y_i \sim Pois(\mu(t_i)).$$

We assume conditional independence in the data, therefore from the Poisson model, we obtain a likelihood. Our parameters are λ_{v_1} and λ_{v_2}. Regarding the elicitation of the parameters prior distribution, we use a Gamma distribution with scale 3 and shape parameter 10. To sample from the posterior, we resort to MCMC using "t-walk" generic sampler [2].

S2.1.3 Posterior distribution and vaccine coverage model results

Estimation of vaccination rates is used to predict vaccine coverage as well as the dynamics of SARS-CoV-2. We displayed the results using the data reported 30 days prior of May 18, 2021. We ran 10,000 iterations of the t-walk and after a burn-in period of 1,000 iterations, the chain seems to be sampling from the equilibrium distribution (i.e., the posterior distribution) (Fig S2C and S2D). Fig S2A and S2B correspond to the marginal posterior distribution for λ_{v_1} and λ_{v_2}. The histograms are reported with 9,000 samples since the first (burn-in) 1,000 are discarded.

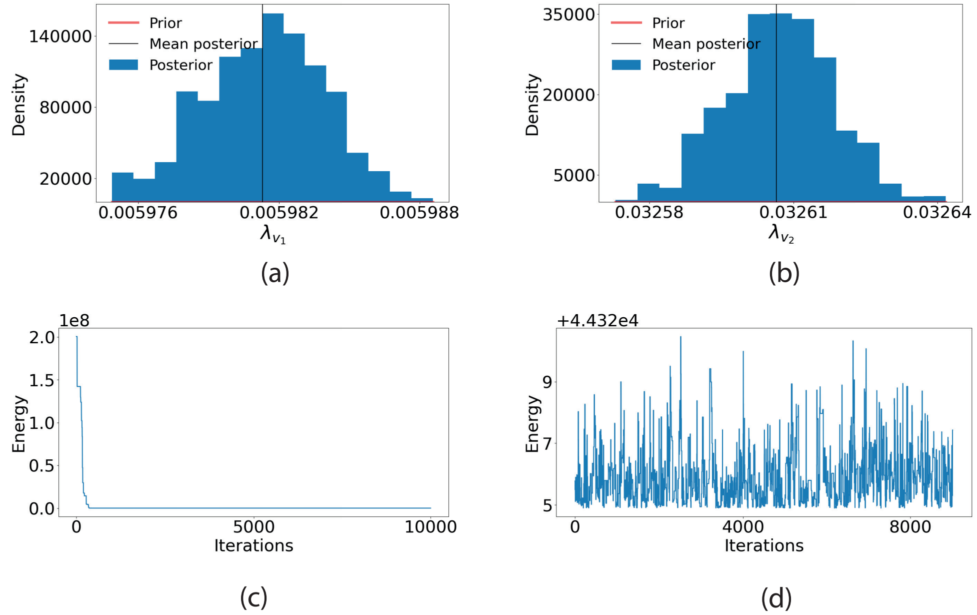

Fig S2. Marginal posterior. After 10,000 MCMC samples, (A)-(B) the marginal posterior distribution for the vaccinate rates λ_{v_1}, λ_{v_2} and its prior distribution (red). (C) Trace plot of the logarithm of the posterior distribution and (D) Trace plot of the logarithm of the posterior distribution without a burn-in of 1,000 iterations.

S2.2 Transmission model

To have more realistic scenarios, we estimate the parameters involved in the dynamical of the SARS-CoV-2 transmission using the Bayesian Sequential Forecasting Method (BSFM) proposed in [1].

S2.2.1 Bayesian Sequential Forecasting Method

The BSFM updates the evolution of the dynamic system from the posterior distribution of both model parameters and state variables as new epidemic records become available. New prior models are defined from the current parameters and state variables posterior distributions on a sliding time window. Within each sliding window, posterior

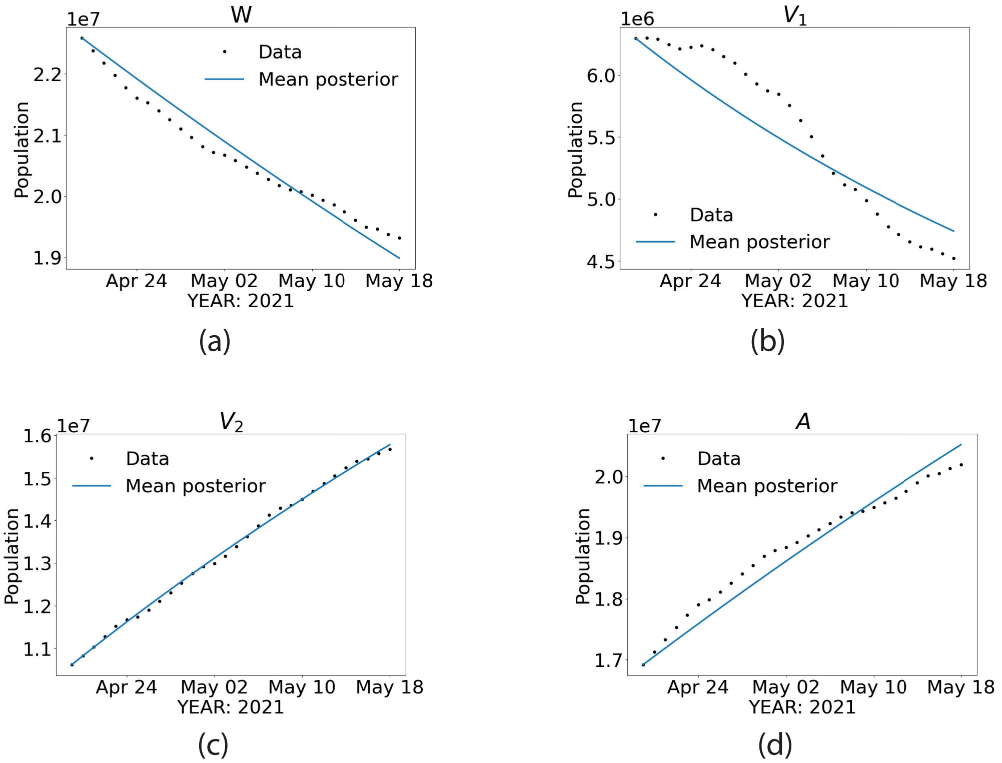

Fig S3. Vaccine coverage model trajectory with median posterior values. (A) people not vaccinated, (B) people with at least one dose, (C) fully vaccinated, and (D) cumulative total of people taking at least one dose at time t.

distributions are computed beyond the available epidemic records to produce the forecasts.

Let $x(t) = (S(t), E(t), I(t), ...)^T$ denote the time–dependent vector of state variables. We shall assume that the epidemic and transmission models are coded in a dynamic system

$$\begin{aligned} \dot{x}(t) &= f(x(t), \theta_k) \\ x(t_k) &= x_k, \end{aligned} \tag{2}$$

where t_k and x_k denote the initial time and state in the forecasting window $[t_k, t_k + L + D + F]$ respectively, θ_k is a vector of model parameters (e.g., contact rate β, effective population size ω, etc.) used to calibrate the model (2), L is the learning period size, F is the number of days to forecast, n is the number of days to move the forecasting window, and D is the number of the delays days. We denoted $p^{(k)} := (x_k, \theta_k)$ as the joint vector of initial conditions and model parameters to be inferred.

The initial forecasting, $k = 0$, is done as a usual Bayesian inference problem, we postulate

- A prior distribution, $\pi_{P^{(k)}}(p^{(k)})$.
- A likelihood, $\pi_{Z^{(k)}|P^{(k)}}(z^{(k)}|p^{(k)})$, where z_k represents epidemic records in t_k to $t_{k+1} + L$ (e.g., confirmed cases, deaths, etc.).
- We use equation (2) and samples obtained through Markov Chain Monte Carlo of

the corresponding posterior distribution, $\pi_{P|Z}(p^{(k)}|z^{(k)})$ to make a probabilistic prediction of $x(t)$ in the forecasting period $t \in [t_k + L + D, t_k + L + D + F]$.

To the next forecasting $k > 0$, we update the forecasting window by setting $t_{k+1} = t_k + n$. The new forecasting window is $[t_{k+1}, t_{k+1} + L + D + F]$. The prior distribution of $p^{(k)} = (x_k, \theta_k)$ is set using the MCMC output of the period $k-1$:

- For the $k-$initial state (x_k), the MCMC output of the state variable $x(t)$ at time $t_0 + nk$ obtained with equation (2) is fitted with a known distribution.
- For the model parameters θ_k, the MCMC output of θ_{k-1} is fitted a known distribution.
- Finally, we set $k \leftarrow k+1$ and repeat the above process to create a new forecast.

The central part of BSFM is that the time dependence of the transmission model parameters is introduced by updating sequential forecasts reported on the history of the outbreak using the posterior distributions as prior distributions for the parameters in the current forecast. Thus, our transmission model becomes a non-autonomous dynamic system, with the same amount of parameters in time and data, capable of capturing changes in outbreak behavior produced by human and virus trend changes.

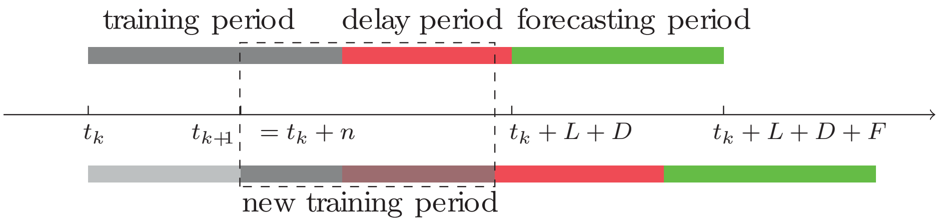

Fig S4. Bayesian sequential data assimilation. The Bayesian filtering method predicts along the dynamical system (2) evaluated in sample points of the posterior distribution $\pi_{P^{(k)}|Z^{(k)}}(p^{(k)}|z^{(k)})$ in the current forecasting window $[t_k, t_k + L + D + F]$.

S2.2.2 Application to California data

The parameter estimation is carried out with model (3), without the vaccination transitions.

$$\begin{aligned}
\dot{S} &= -\lambda S \\
\dot{E} &= \lambda S - f\sigma E - (1-f)\sigma E \\
\dot{O} &= f\sigma E - \gamma_o O - (1-g)\gamma_o O \\
\dot{U} &= (1-f)\sigma E - \gamma_u U \\
\dot{R} &= (1-g)\gamma_o O + \gamma_u U \\
\dot{D} &= \gamma_o g O.
\end{aligned} \tag{3}$$

Using data of confirmed cases and deaths, we estimate the contact rate (β), the proportion of the effective population (ω), the fraction of individuals infected that are deceased (g), and the initial conditions for all compartments, except for the susceptible ones, which are set as $S(t_0) = \omega \cdot N - (E(t_0) + O(t_0) + U_(t_0) + R_(t_0)) + V_1(t_0) + V_2(t_0)$.

We consider records of confirmed cases and deaths from January 25, 2020, until May 10, 2021. These data are smoothing using a weekly moving average, which is calculated for each day by averaging the values of that day and the six days before. This approach helps prevent significant events (such as changing reporting methods) from skewing the data. Using the BFSM for California data, we forecast every eight days with the most recent data. Fig S5 shows the forecasts from December 6 to May 18, 2021, for both confirmed cases and deaths. Fig S6 shows the trajectory of ω, θ, and g for the pandemic period. The public response to long-term mitigation measures for the pandemic is reflected in the evolution of β and ω parameters.

The β contact rate takes a high value at the beginning of the pandemic but declines after the first intervention in California (March 12, 2021) and stabilizes around 0.35. The value of ω has shown greater variability over time. Like β, it takes high values at the beginning of the pandemic and declines with the first intervention carried out in California in March. In July and December, we observed an increase in the values of this parameter that coincides with the waves that California had in the same months. The proportion of observed deaths (g) decreases with time, probably due to experience gained over time in caring for patients and expanding hospital capacity. After a while, this value stabilizes and remains close to 0.03.

For the vaccination model (Eq 1 main text), we use the posterior median in the last forecast for initial conditions, contact rate β, effective population ω, and proportion of deaths g. We take the initial condition for vaccination like the current vaccine and redefine $S(t_0) = \omega N_0 - V_0 - E_0 - U_0 - O_0 - R_0 - D_0$.

S2.2.3 Prediction interval coverage

We have the probabilistic one-, two-, three-, and four-week ahead forecasts of the total number of confirmed cases and deaths due to COVID-19 from January 13, 2020, to May 18, 2021, and every eight days after that for California. We evaluated our forecast error with the calibration of the prediction interval coverage (80% and 50%). The prediction interval coverage is calculated by determining the frequency with which the prediction interval contained the eventually observed outcome. We do this for all prediction intervals calculated from January 13, 2020, to May 18, 2021, and calculate the average of these. In a model that accurately characterizes uncertainty, the prediction interval level will correspond closely to the frequency of eventually observed outcomes that fall within that prediction interval. For example, finally observed values should be within the 50% prediction interval approximately 50% of the time.

Table S1. Observed prediction interval coverage.

	Cases				Deaths			
	Observed Prediction Interval Forecast Horizon (weeks ahead)				Observed Prediction Interval Forecast Horizon (weeks ahead)			
Measure	week 1	week 2	week 3	week 4	week 1	week 2	week 3	week 4
50% Coverage	0.60	0.56	0.52	0.49	0.59	0.64	0.66	0.67
80% Coverage	0.83	0.80	0.76	0.73	0.94	0.94	0.93	0.91

The observed prediction interval coverage for confirmed cases / deaths from January 28, 2020 to May 18, 2021 in California was calculated by taking the average coverage of the prediction intervals observed in the sequential prediction for California.

The forecasts were well-calibrated, with prediction intervals covering the observed data with the expected frequency (Table S1). The 50% prediction intervals captured 48-60% of observations for all forecast horizons for confirmed cases and 58-66% for deaths. The 80% prediction intervals captured only 73-83% of confirmed cases and

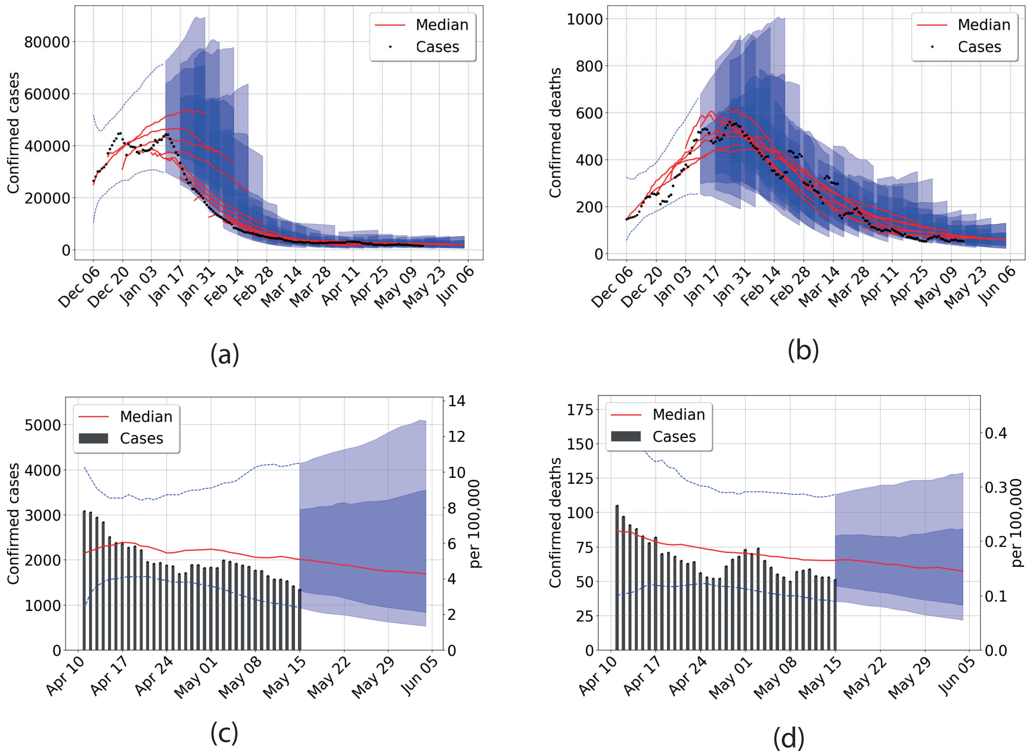

Fig S5. California outbreak analysis. Data from April 8 to May 18, 2021 is used. (A) Confirmed cases (B) Confirmed deaths. Central red lines indicate the median incidence forecast. The darker shaded region indicates the interquartile forecast range, and the lighter shaded region indicates the 5–95th percentile range. All displayed forecast duration's are ten days from the point of prediction. California total population 39,512,223.

91-94% for deaths. The intervals were better calibrated for confirmed death due that the record of Covid-19 deaths is more reliable than records of confirmed cases. The last one depends directly on the number of tests applied.

S3 Other results

These results are similar to the results described in the main text, but instead of reducing the current vaccination rate by 30%, we consider the scenario where this value decreases by 60%.

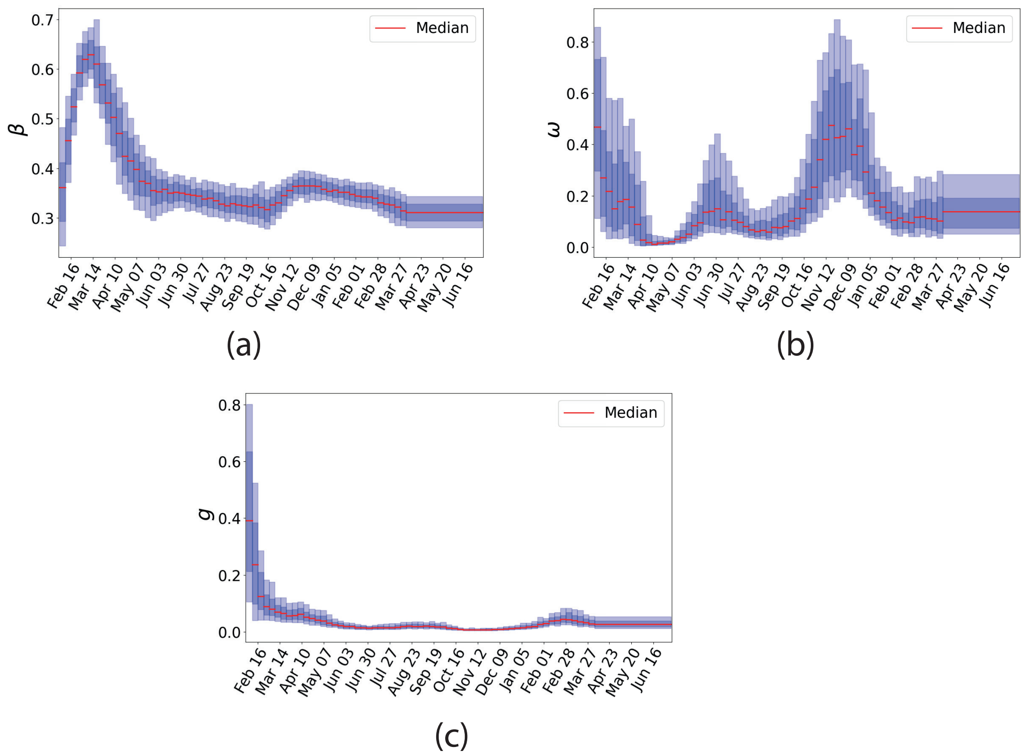

Fig S6. The trajectory of the parameters during the pandemic period. From left to right, contact rate after lockdown (β), proportion of the effective population (ω), and the fraction of infected dying (g). Central red lines indicate median incidence forecast. Darker shaded region indicates forecast interquartile range, and lighter shaded region indicates 5–95th percentile range.

Table S2. Parameters values for the baseline scenario correspond to the posterior median value $\beta_{base} = 0.31$, $\omega_{base} = 0.12$, $\lambda_{v1} = 0.00598$, $\lambda_{v2} = 0.032$; $\beta = 0.4, 0.5$, and $\omega = 0.3, 0.5$ were selected according to historical data in CA (Fig. S6).

	Vaccination rate assumptions					
	Current vaccination rate	Current vaccination rate decrease 60%	Current vaccination rate increase 30%	Current vaccination rate	Current vaccination rate decrease 60%	Current vaccination rate increase 30%
Parameters	Increase or prevention percentage in cases 15 days after opening			Increase or prevention percentage in deaths 15 days after opening		
$\beta_{base} = 0.31$	11429*	80.4	-26.1	429*	50.7	-17.9
$\beta_1 = 0.4$	21.8	122.6	-10.5	4.4	61.1	-14.6
$\beta_2 = 0.5$	48.5	172.5	8.4	9.6	73.5	-10.8
$\omega_{base} = 0.12$	9829*	77.5	-25.6	402*	52.0	-16.4
$\omega_1 = 0.3$	51.5	139.1	22.7	12.2	65.8	-5.1
$\omega_2 = 0.5$	68.9	159.9	37.6	15.6	69.2	-2.7

*Base scenario values (total cases and deaths between June 15 and June 30, 2021). All percentages are calculated based on these values.

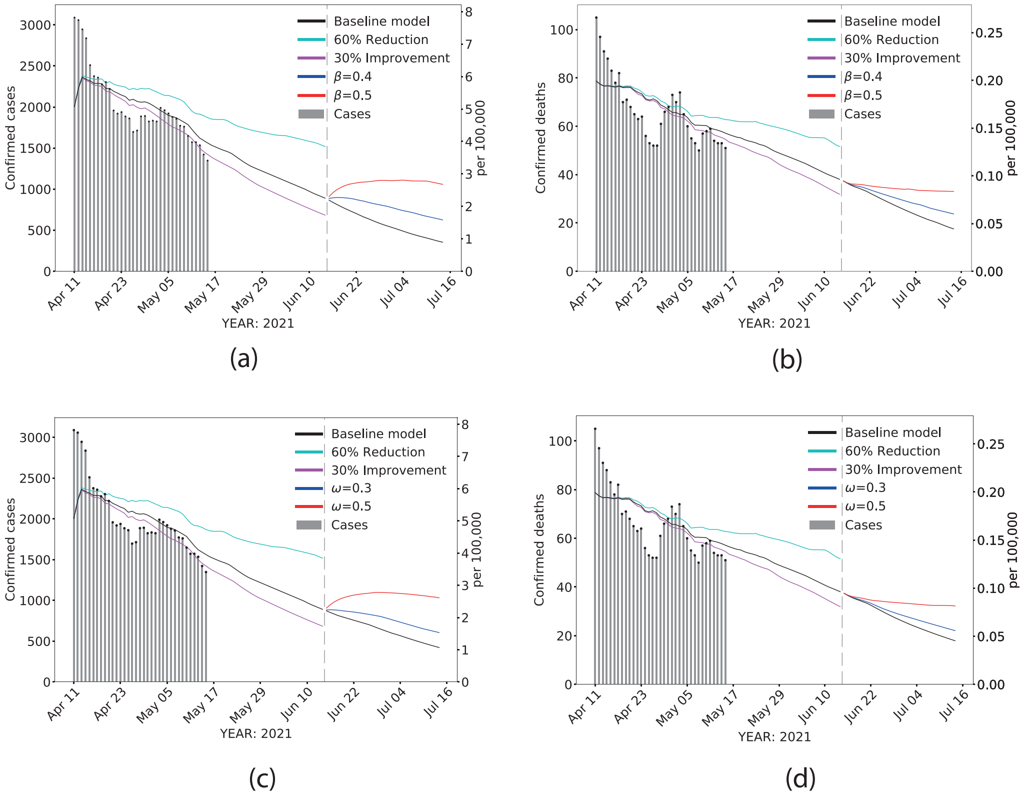

Fig S7. Scenarios. The dashed vertical line indicates the opening day, June 15. Reported data are shown in gray (bars). The black line corresponds to the baseline scenario, the cyan line corresponds to the projection assuming a 60 % decrease in the current vaccination rate, and the magenta line corresponds to the projection assuming a 30% increase in the current vaccination rate. After June 15, the red, blue, and black lines correspond to the projection with the different values of ω, cases (A) and deaths (B) and changes in β (C) cases and (D) deaths, with the current vaccination rate.

References

1. Daza-Torres ML, Capistrán MA, Capella A, Christen JA. Bayesian sequential data assimilation for COVID-19 forecasting. Epidemics. Forthcoming.

2. Christen, J Andrés and Fox, Colin and others A general purpose sampling algorithm for continuous distributions (the t-walk). Bayesian Analysis. 2010;5(2): 263–282.
